# Low temperature deformation behavior of an electromagnetically bulged 5052 aluminum alloy

**DOI:** 10.1038/srep29973

**Published:** 2016-07-18

**Authors:** Zu Li, Ning Li, Duzhen Wang, Di Ouyang, Lin Liu

**Affiliations:** 1 School of Materials Science and Engineering and State Key Laboratory of Material Processing and Die & Mould Technology, Huazhong University of Science and Technology, 430074 Wuhan, PRC

## Abstract

The fundamental understanding of the deformation behavior of electromagnetically formed metallic components under extreme conditions is important. Here, the effect of low temperature on the deformation behavior of an electromagnetically-bulged 5052 aluminum alloy was investigated through uniaxial tension. We found that the Portevin-Le Chatelier Effect, designated by the serrated characteristic in stress-strain curves, continuously decays until completely disappears with decreasing temperature. The physical origin of the phenomenon is rationalized on the basis of the theoretical analysis and the Monte Carlo simulation, which reveal an increasing resistance to dislocation motion imposed by lowering temperature. The dislocations are captured completely by solute atoms at −50 °C, which results in the extinction of Portevin-Le Chatelier. The detailed mechanism responsible for this process is further examined through Monte Carlo simulation.

Fascinating attention was paid to the light weight design in the past decades due to the challenge in environmental sustainability, energy crisis and climate change. The substitution of conventional structural metals such as steel and cast iron by lightweight magnesium and aluminum alloys thus becomes crucial in lightweight manufacturing. However, aluminum alloys have inherent poor formability in contrast to conventional mild steels, especially by traditional cold forming process. This has seriously limited the extensive application of aluminum alloys in modern industries. Electromagnetic forming (EMF)^1^ is a revolutionized manufacturing technique based on high velocity forming, which brings a plethora of advantages such as improved formability^2^,^3^,^4^, contactless forming^5^, reduced springback^6^,^7^, suppressed wrinkling^8^ and high repeatability^5^. These characteristics altogether enable EMF to rival most conventional mechanical processing techniques of aluminum alloys.

In the EMF of aluminum alloys, microstructural evolution has become one of major concerns due to its dominant role in determining the formed component’s properties. Comparing with the alloy underwent quasi-static deformation, Risch *et al*.^9^ reported that the AA5182-type aluminum alloy after EMF exhibits small grain size and abundant dislocation motion, owing to the high strain rate deformation. Liu *et al*.^10^ also found that the high-velocity electromagnetic deformation facilitate cross-slip of dislocations, high dislocation density, and uniform dislocation configuration in 5052 aluminum alloy, which significantly promotes the plastic strain and strength. Li *et al*.^11^ revealed a textural evolution from Rotated Cube towards Cube and Goss & Rotated Goss under EMF, accompanied with the increased dislocation density and intra-granular misorientations. In addition to the effect of high strain rate, plastic strain has been correlated to the microstructural evolution. For instance, Bach *et al*.^12^ revealed that with increasing plastic strain, dislocation density in pure aluminum increases, followed by formation of highly localized tangles into cell-like structures with different cell wall thicknesses until sub-grains finally evolve. Jiang *et al*.^13^ observed similar phenomenon in an electromagnetically bulged pure copper.

In our recent research^14^, we systematically investigated microstructure, texture and mechanical properties of annealed 5052 aluminum alloy tubes after EMF. The interesting finding is that the EMF induces a significant increase in yield strength and fracture strength but decrease in fractural elongation due to the strain hardening effect resulting from the increase in dislocation density and the formation of high density dislocation bands. We also probed the fatigue behavior of the electromagnetically bulged 5052 aluminum alloy through tensile-tensile fatigue testing. We found that the electromagnetically bulged specimen exhibits enhanced fatigue strength as depicted by the S-N (maximum stress *vs.* the number of cycles until failure) curves, as compared to the original (un-deformed) aluminum alloy^15^. Furthermore, these electromagnetically-formed aluminum alloy components are usually applied in aircraft which is typically exposed in extreme conditions such as low temperatures (e.g., 50 degrees below zero). Therefore, the low temperature deformation behavior and the related mechanism of these electromagnetically-formed aluminum alloy components are of tremendous interest, whereas the research has rarely been reported thus far. In the present work, the 5052 aluminum tubes are electromagnetically bulged and the effect of low temperature on the deformation behavior of the bulged aluminum alloy is investigated through uniaxial tension. We found that the Portevin-Le Chatelier (PLC) Effect known as serrated features in stress-strain curves continuously decays until completely disappears with decreasing temperature. This phenomenon can be understood in-depth on basis of the interaction of solute atoms and dislocations under various temperatures. Our findings deliver a fundamental understanding to the low temperature deformation behavior of electromagnetically bulged aluminum alloys.

## Results

### Temperature dependent Portevin-Le Chatelier Effect

**Figure 1:**
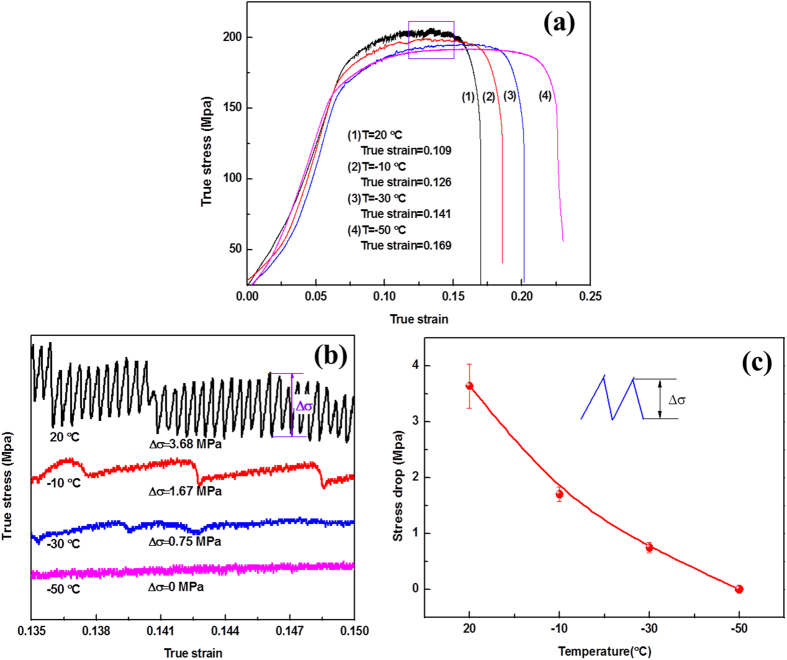
(**a**) True stress-strain curves for the EMF-bulged 5052 aluminum alloys deformed at various temperatures; (**b**) magnification of the square signed in (**a)**; (**c**) the corresponding statistical results of stress drop with temperatures.

Figure 1a illustrates true stress-strain curves of the electromagnetically bulged 5052 aluminum alloy under low temperature (ranging from 20 °C to −50 °C) tension with a stain rate of 1 × 10^−3^ s^−1^. On the whole, the plastic strain increases with the reduction of temperature, for example, at ambient temperature, the true strain (*ε*) is about 0.109, while *ε* increases up to 0.126 and 0.141 when temperature decreases to −10 °C and −30 °C, respectively. This tendency becomes more conspicuous for the specimen tested at −50 °C, wherein the true strain advances to about 0.169. The similar phenomenon can also be observed in strength (*σ*) of the electromagnetically bulged 5052 aluminum alloy, *σ* decreases with reducing temperatures, exhibits a softening effect. From Fig. 1a, we also notice that most curves exhibit continuous serrated flow characterization in a certain range of plastic strains, these serrated flow features are well known as the Portevin-Le Chatelier (PLC) Effect^16^,^17^,^18^,^19^.

In order to further investigate the effect of temperature on the deformation behavior of the electromagnetically bulged 5052 aluminum alloy, the stress-strain curves at a certain range of strains (from 0.135 to 0.150 located in a relative stable state) are magnified, as depicted in Fig. 1b. To clearly distinguish each stress-stain curve while presenting all of them in the same plot, some of the curves were shifted along the stress axis. It is worth noting that the magnitude of serrations decays with reduction of temperature from 20 °C to −30 °C, and finally disappears at −50 °C. To distinguish quantifiably the difference of PLC effect caused by the variation of experimental temperatures, stress drop (*Δσ*, the difference between the maximum stress and minimum stress in each serration, as described in Fig. 1b) was analyzed statistically, and the statistical result of stress drop with temperatures is shown in Fig. 1c. The stress drop is about 3.68 MPa at ambient temperature (20 °C), whereas it decreases linearly to about 1.67 MPa and 0.75 MPa when the temperature reduces to −10 °C and −30 °C, respectively, Finally, *Δσ* equates to zero at −50 °C, indicates temperature dependent PLC effect.

### Temperature dependent deformation mechanism

**Figure 2:**
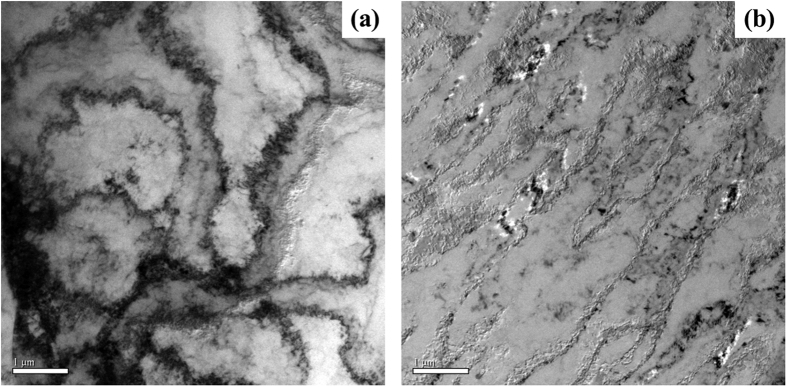
Dislocation structures of EMF-bulged 5052 aluminum alloys deformed at 20 °C (**a**) and −50 °C (**b**).

To probe possible effect of low temperature on deformation mechanism, electromagnetically-bulged 5052 aluminum alloy after low-temperature tension were characterized by transmission electron microscope (TEM), and the results are presented in Fig. 2. Here the bright field images of specimens tested under 20 °C and −50 °C are selected for further analysis. From Fig. 2a, dislocation configuration is characterized by irregular dislocation cells mixed with loose dislocation tangles, indicates that the cross slipping dominates dislocation motion in the electromagnetically formed aluminum alloy at ambient temperature. However, when the experimental temperature decreases to −50 °C, parallel dislocation bands can be clearly observed (see Fig. 2b), indicates planar slipping of dislocation^20^.

## Discussion

In general, the serrated characteristics in stress-strain curve indicate the occurrence of heterogeneous deformation. In this case, plastic stains are localized in very small regions such as shear bands or Portevin-Le Chatelier (PLC) bands, which finally causes the catastrophic fracture of the sample^20^. Therefore, it can be well understood that the decay of serrations with decreasing temperature from 20 °C to −50 °C corresponds to the increase of plastic strains, as depicted in Fig. 1a.

This temperature dependent PLC effect can be understood fundamentally on the basis of the interaction between solute atoms and dislocations. Dislocation usually operates at yield stress wherein the lattice elastic distortion generates stress field (P) around dislocations^21^,^22^, causing enthalpy change *PΔV* (*ΔV* is solute misfit volume). In this case, solute atoms diffuse to dislocations in order to release stress and minimize system energy^17^,^21^,^23^. These solute atoms around dislocations are usually called “Cottrell atmosphere”^24^ that usually pins dislocations, which causes an increase of frictional stress^25^ around solute atoms, and impedes dislocation motions. Therefore, high external stress is required to overcome this pinning energy, results in an increase of stress. Once external stress subjugates pinning energy, dislocations extricate the constraint of solute atoms, causing an instantaneous stress drop. The continuous process of pinning-shake off, leading to the serrated characteristics in stress-strain curves, known as the Portevin-Le Chatelier (PLC) effect^17^,^26^,^27^ as observed in Fig. 1. The above interactions between solute atoms and dislocations, affected inherently by temperature, which can be understood in-depth according to the diffusion rate (*D*) of solute atoms based on the Arrhenius equation^28^:
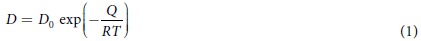
in which *D*_*0*_ = 6.23 × 10^−4^ m^2^/s is the diffusion constant, *Q* = 1.19 eV is the diffusion activation energy, *R* = 8.314 J/(mol·K) is the gas constant^28^ and *T* is the absolute temperature. From equation (1), temperature is the decisive parameter for diffusion rate of solute atoms. Therefore, diffusion rates at various temperatures can be calculated and results are listed in Table 1, from which *D* is 2.29 × 10^−24^ m^2^/s under room temperature uniaxial tension, but it decreases monotonously from 1.07 × 10^−26^ m^2^/s to 1.42 × 10^−28^ m^2^/s with reducing temperature from −10 °C to −30 °C. This tendency becomes conspicuous at −50 °C (*D* = 8.81 × 10^−31^ m^2^/s), indicates serious difficulties of solute atoms diffuse at low temperature.

**Figure 3:**
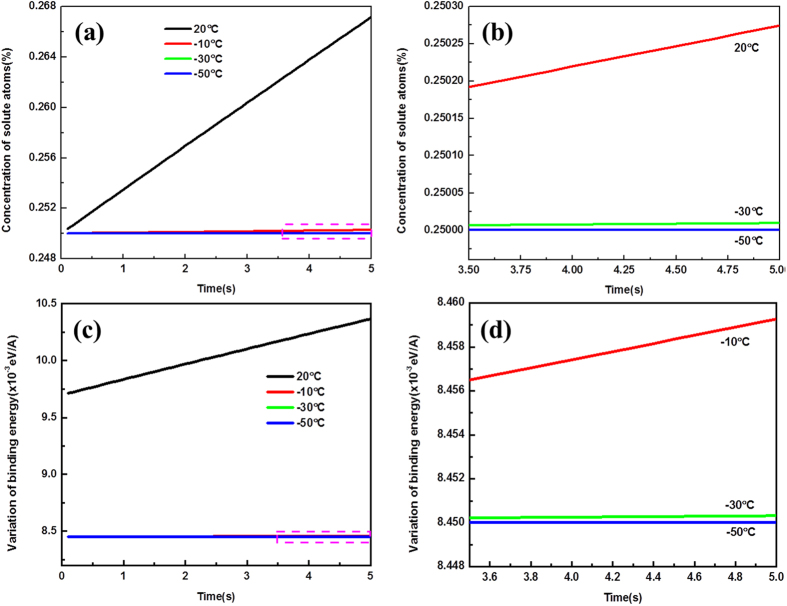
(**a,b**) The calculated concentration of solute atoms around the dislocation; (**c,d**) variation of binding energy between solute atoms and dislocation per unit length.

According to “cross-core diffusion” model, the concentration of solute atoms (*c*(*t*)) around the dislocation can be expressed as^23^
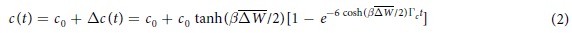
in which *C*_*0*_ = 2.5% is the bulk solute concentration^20^, *ΔW* = 0.13 eV is the average binding energy difference, *β* = *1/kT* (*k* = 1.38 × 10^−23^ J/K is the Boltzmann constant and *T* is the absolute temperature), 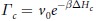 is the transition rate^23^ (*v*_*0*_ = 3.8 × 10^13^ s^−1 ^28 is the attempt frequency and Δ*H*_*c*_ = 0.97 eV^23^ is the average activation enthalpy). By applying the data listed in Table 2, the concentration of solute atoms around dislocations can be calculated and plotted in Fig. 3a. It is shown that *c*(*t*) around dislocations decreases with the reduction of temperature, especially from 20 °C to −10 °C, and this tendency continues at low temperatures from −10 °C to −50 °C, as depicted in Fig. 3b. The variation of solute atoms concentration (Δ*c*(*t*)) then induces the *c*hange of binding energy (Δ*E* (*t*)) between solute atoms and dislocations per unit length, here^23^
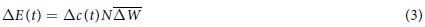
in which 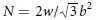 is the number of solute atoms around the dislocation with a width *w* ≈ 21 Å^23^ *b* = 0.285 nm^29^. According to equation (3), ΔE (t) are calculated and the result is depicted in Fig. 3c. Conspicuous decrease of binding energy can be detected when the temperature decreasing from 20 °C to −10 °C, and then this reduction becomes gradual from −10 °C to −50 °C (Fig. 3d), which responsible for the serrated characteristics in stress-strain curves as observed in Fig. 1b.

**Figure 4:**
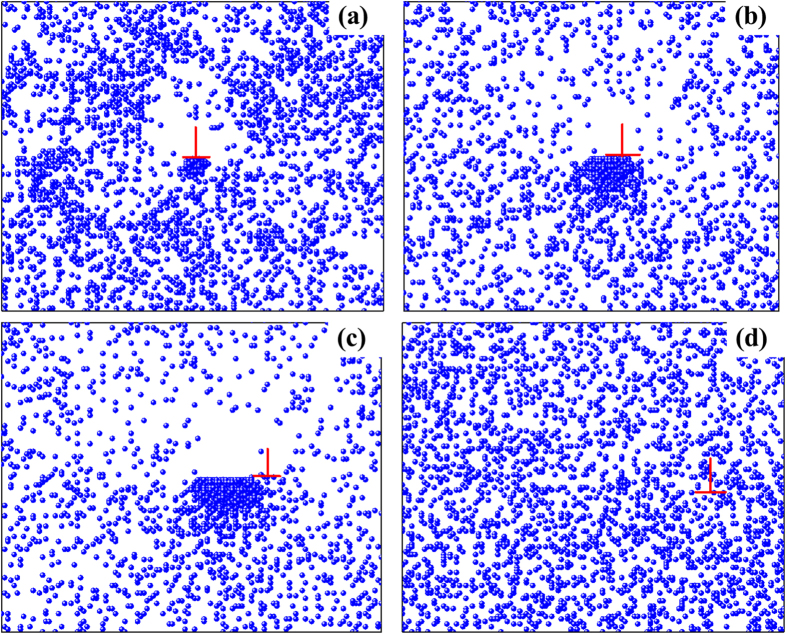
The simulated distribution of solute atoms around dislocation under 20 °C at various moments: (**a**) 10^6^ (1/Γ), (**b**) 10^7^ (1/Γ), (**c**) 10^8^ (1/Γ), (**d**) 5 × 10^8^ (1/Γ).

The micro-mechanism of the present phenomenon can be further understood through Monte Carlo simulation. Here, the simulated results at two typical temperatures (namely 20 °C and −50 °C) are selected for analysis. Figure 4 shows the interactions between solute atoms and dislocations at various time when *T* = 20 °C. It is clear that when *t* = 1.0 × 10^6^ (1/*Г*), partial solute atoms gather below the dislocation (Fig. 4a), and then, the quantity of agminated solute atoms rises synchronously with increasing time (Fig. 4b,c), which forms Cottrell air ass and increases deformation stress. Dislocations finally shake off the shackle of solute atoms (Fig. 4d) and move freely due to the effect of thermal activation^24^,^30^, results in stress dropping. The continuous process of pinning-shake off, results in PLC effect as observed in Fig. 1b 31,32.

**Figure 5:**
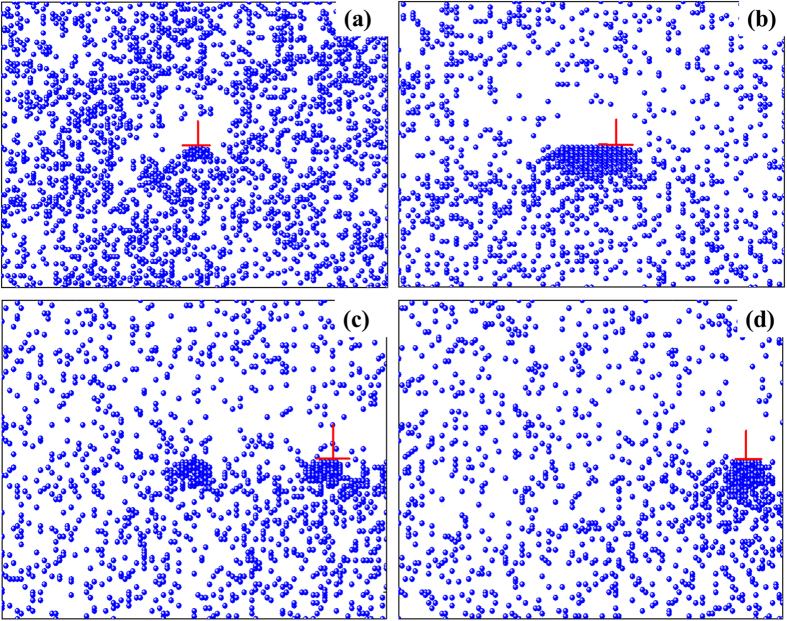
The simulated distribution of solute atoms around dislocation under −50 °C at various moments. (**a**) 10^6^ (1/Γ), (**b**) 10^7^ (1/Γ), (**c**) 10^8^ (1/Γ), (**d**) 5 × 10^8^ (1/Γ).

The interaction between solute atoms and dislocations at −50 °C is depicted in Fig. 5. Due to the decay of diffusion coefficient with dropping temperature (Table 1), the motion of dislocations becomes slow. Therefore dislocations hardly shake off the constraint of solute atoms. In this case, solute atoms and dislocation move together, as illustrated in Fig. 5d, which annihilates the PLC effect in stress-strain curve^31^,^33^,^34^,^35^.

**Figure 6:**
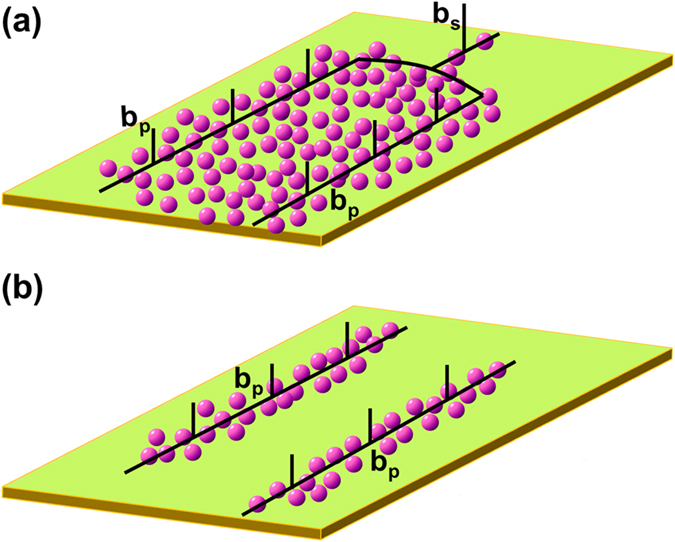
Dissociation of a perfect dislocation into two Shockley partial dislocations: (**a**) 20 °C, the size solute atmosphere is large enough to surround both partials; (**b**) −50 °C, the concentration of solute atoms around the partials is low.

Lowering the temperature also includes a transition of slipping mode from cross slip to planar slip as depicted in Fig. 2. As discussed above, the interaction between solute atoms and dislocations varies with temperatures. As shown in Fig. 6, in general, a perfect dislocation dissociates into two Shockley partial dislocations, expressed as:
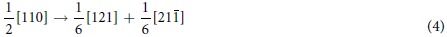


At ambient temperatures, the concentration of solute atoms around dislocations is high, and the size of solute atmosphere (i.e. Cottrell air ass) is large enough to surround both partials, namely, two partials are full of solute atoms (Fig. 6a). In this case, partials can join together easily to form a perfect dislocation without escaping from “solute atmosphere”^25^, which facilitates the cross slip and forms irregular dislocation cells mixed with loose dislocation tangles as observed in Fig. 2a. While at −50 °C, the concentration of solute atoms around the partials is low and can’t embrace partials (Fig. 6b), therefore partials have to shake off “solute atmosphere” to form a perfect one, which hinders the cross slip and tends to parallel slip.

## Conclusions

In summary, the effect of temperature on deformation behavior and microstructure of 5052 aluminum alloy was investigated systemically. The conclusions can be drawn from experimental results as follows:

1. The serrated characteristics in stress-strain curves (*i.e.* PLC effect) decay with the reduction of temperatures, and annihilate at −50 °C. At ambient temperature, the continuous process of pinning-shake off between solute atoms and dislocations, responsible for the PLC effect. While at low temperature, the interaction between solute atoms and dislocations decays, and even move together at −50 °C, causes the annihilation of PLC effect.
2. The decreasing temperature also results in a transition of slipping mode from wave slip (20 °C) to planar slip (−50 °C). At ambient temperatures, the size solute atmosphere is large enough to surround both partials, and partials can join together easily to form a perfect dislocation without necessary escaping from “solute atmosphere”, which facilitates the cross slip. While at low temperature of −50 °C, the concentration of solute atoms around the partials is low, the partials have to shake off “solute atmosphere” to form a perfect one, results in parallel slipping.

## Experimental Methods

### Electromagnetic bulging

The as-received 5052 aluminum alloy tube with inner diameter of 48 mm and thickness of 1 mm was supplied by Northeast Light Alloy Co., Ltd., China. The nominal chemical composition (wt. %) of the alloy is listed in Table 2. It is notable that Mg, Si and Fe are the solute atoms in the aluminum alloy.

**Figure 7:**
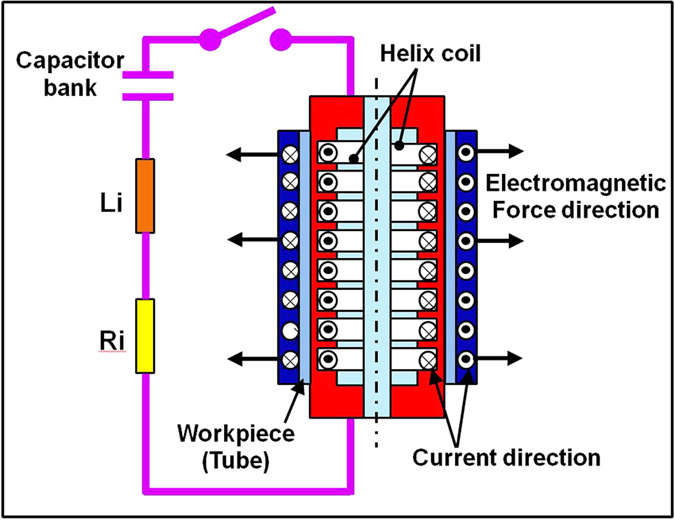
The scheme of the electromagnetic bulging of tube.

The 5052 aluminum alloy specimens with length 200 mm used for electromagnetic bulging were fabricated by wire-cutting from the as-received tubes. In order to diminish pre-extrusion induced residual stress and dislocations in the original tube, the tube specimens were then annealed at 653 K for 120 minutes before electromagnetic bulging. The experiments were performed on the self-build EMF system (EMF 30) at Harbin Institute of Technology. The scheme of the experimental apparatus is depicted in Fig. 7, wherein a 22-turn helix coil with an outer-diameter of 47 mm and effective length of 190 mm was used to bulge the tubes. From Fig. 7, the system consists of a capacitor bank connected to the helix coil which was placed coaxially inside the aluminum alloy tube. The bulging of the tube was designed to be free expansion without fixtures. The coil was covered homogenously with polyimide films to provide insulation and to ensure a good coaxiality between coil and tube (the coil was wrapped uniformly with polyimide film so that the outer diameter of the coil can fit well with the tube). Upon discharging the capacitor, time-varying currents flowed through the coil and generated a transient magnetic field, which induced an eddy current in the aluminum tube and generated an opposing transient magnetic field. The interaction of these two magnetic fields created large repulsive Lorentz body forces to drive a rapid plastic deformation of aluminum alloy tubes. In the present experiments, a discharge voltage of 10 kV was applied in the electromagnetic bulging, and at least 3 times repeated trials were carried out to ensure the reliability.

### Low temperature uniaxial tensile texting and microstructure characterization

**Figure 8:**
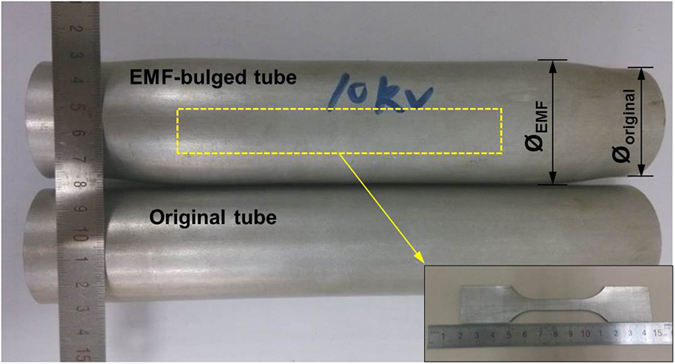
Photographs of the original and electromagnetically bulged tubes, the insert shows the tensile specimen cut from the yellow square of the EMF-bulged tube.

Figure 8 shows photographs of the electromagnetic bulged aluminum alloy tube as compared with the original tube. It is evident that the diameter of the electromagnetically-bulged tube becomes noticeably large after bulging (Ø_EMF_ > Ø_Original_ as shown in Fig. 8), the uniform plastic deformation indicates homogeneous distribution of microstructure. In order to probe the possible variation of mechanical properties induced by electromagnetic bulging, specimens in both original and bulged states were fabricated by wire-cutting along the longitudinal direction of the tubes (see Fig. 8), wherein the machined dog-bone shape samples with gauge length of 25 mm and width of 8 mm. The uniaxial tension experiments were conducted on an electronic universal testing machine (RGM-4050) at strain rate of 1 × 10^−3^ s^−1^. In order to probe the effect of low temperature on the deformation behavior of the electromagnetic-formed aluminum alloy, various experimental temperatures ranging from 20 °C, −10 °C, −30 °C to −50 °C were applied.

Microstructures of the electromagnetically-bulged aluminum alloys after uniaxial tension at various temperatures were characterized by TEM (FEIT ecnai G20). TEM thin foils were first mechanically grinded to about 50 μm thick, followed by a twin-jet polishing method in a solution of 30% nitric acid and 70% methanol at a voltage of 20 V and at the temperature of −20 °C.

### Monte Carlo simulation

**Figure 9:**
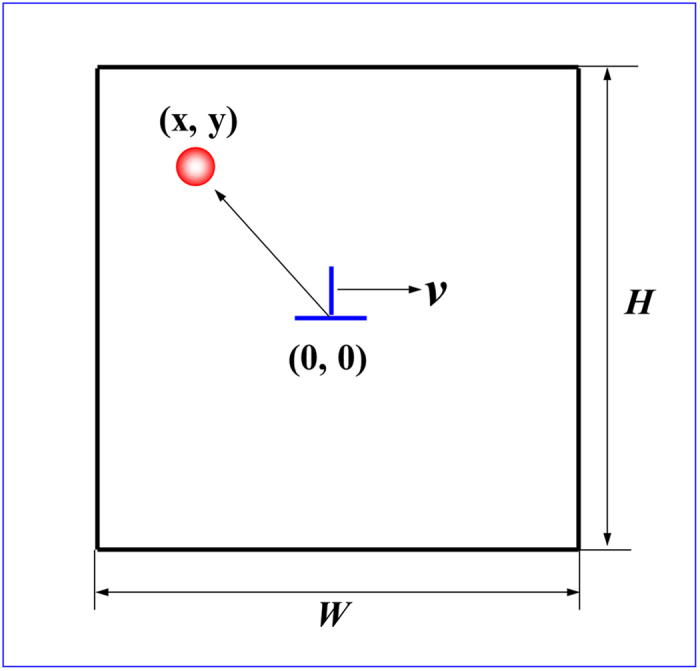
Schematic illustration of the simulation cell.

As mentioned above, there are some solute atoms (such as Mg, Si and Fe shown in Table 2) in 5052 aluminum alloy, which usually causes the lattice distortion and generates stress field (*P*) around dislocations, the enthalpy change (*ΔE*) can be defined as^36^,
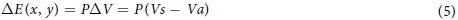
in which *V*_*s*_ and *V*_*a*_ are the volumes of the solute and solvent atoms *ΔV* = 6.3 × 10^−30^ m^3 ^30. *P* is the pressure field associated with the position of the solute atoms that correlated to the dislocation (as depicted in the coordinates of Fig. 9), expressed as^29^
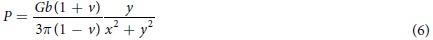
where *G* = 27 GPa is the isotropic shear modulus, *ν* = 0.32 is the Poisson’s ratio of the 5052 aluminium alloy^37^,^38^ and ***b*** = 0.285 nm is the Burgers vector of dislocation^29^. To better analyze dislocation movement and interaction with solute atoms, a single dislocation is introduced in present simulation^36^,^37^. The edge dislocation is restricted to glide on a single slip plane and no climb is possible, using periodic boundary conditions. The solute atom is free to move in any direction within the two-dimensional square lattice. We calculate the change in energy of the system, Δ*E* (equation 5), If Δ*E* < 0, the system energy decreases, the attempted move is accepted; If Δ*E* > 0, the system energy increases, accept the mobile at a certain probability^39^.

Monte Carlo simulation of the unit size is 200 *b* × 200 *b*, namely high *H* = 200 *b*, width *W* = 200 *b*, *b* is the size of Burgers vector in 5052 aluminum alloy. The edge dislocation at the center of the unit, regarded as the origin of coordinate system, as shown in Fig. 9. Assume that each lattice point is occupied only by one solute atom (such as Mg)^38^. The time reported in units of the minimum time required for the dislocation to advance by one Burgers vector, 1/*Г*^36^,^40^.

## Additional Information

**How to cite this article**: Li, Z. *et al*. Low temperature deformation behavior of an electromagnetically bulged 5052 aluminum alloy. *Sci. Rep.* **6**, 29973; doi: 10.1038/srep29973 (2016).

## Figures and Tables

**Table 1:** Diffusion coefficient of Mg in Al(D_Mg/Al_) at different temperature.

T(°C)	−50	−30	−10	20
D_Mg/Al_(m^2^/s)	8.81 × 10^−31^	1.42 × 10^−28^	1.07 × 10^−26^	2.29 × 10^−24^

**Table 2:** 

**Mg**	**Si**	**Fe**	**Cu**	**Mn**	**Ti**	**Al**
2.0 – 2.8	0.4	0.4	0.1	0.15 – 0.4	<0.1	Balance
Nominal composition of the 5A02 aluminum alloy (in wt.%).						
